# Speech-language therapy and occupational therapy for patients with mild cognitive impairment and dementia: a retrospective cohort study using German health claims data

**DOI:** 10.1186/s12913-025-13149-y

**Published:** 2025-08-05

**Authors:** Fiona Dörr, Daniela Holle, Bashar Morouj, Dominik Obermüller, Sascha Sommer, Markus Wübbeler, Kerstin Bilda

**Affiliations:** 1 Department of Nursing, Midwifery and Therapeutic Sciences, Bochum University of Applied Sciences. Location Health Campus, Gesundheitscampus 6-8, Bochum, 44801 Germany; 2 InGef-Institute for Applied Health Research Berlin GmbH, Otto-Ostrowski- Str. 5, Berlin, 10249 Germany

**Keywords:** Dementia, Mild cognitive impairment, Cognitive interventions, Speech-language therapy, Occupational therapy, Allied health professionals, Claims data, Health services research

## Abstract

**Background:**

Dementia is a complex, multifactorial syndrome characterised by cognitive decline and impaired daily functioning, representing a major risk factor for long-term care dependency. As the prevalence of dementia will increase due to demographic change, healthcare systems face growing challenges, including timely diagnosis, equitable access to care, and managing the rising demand for health services. Speech-language therapy (SLT) and occupational therapy (OT) can help maintain cognitive function and quality of life, particularly in the early stages of dementia or mild cognitive impairment (MCI). However, their utilisation in Germany remains poorly understood.

**Objective:**

This study evaluates the utilisation and prescription patterns of SLT and OT among people with dementia or MCI and explores factors associated with therapy use, based on German claims data.

**Methods:**

A retrospective cohort study was conducted using routinely collected data from the research database of the Institute for Applied Health Research Berlin (InGef), including anonymised health records of 5 million individuals in Germany. The dataset covered the period from 2017 to 2022. Incident cases of dementia or MCI were tracked for two years following diagnosis to assess therapy use and prescription patterns. Different facets associated with therapy use were analysed using multivariable logistic regression.

**Results:**

A total of 63,496 individuals (58% female, 42% male) were included (81.8% with dementia, 18.2% with MCI). Of these, 4.2% received SLT and 10.3% received OT (at least one prescription within the two-year follow-up period).

Male sex (Odds Ratio [OR] 1.24, 95% confidence interval [CI] 1.09–1.40) and certain dementia types were significantly associated with higher odds of receiving SLT compared to individuals with Alzheimer’s disease (AD): dementia classified elsewhere (OR 3.34, 95% CI 2.46–4.53), vascular dementia (OR 1.71, 95% CI: 1.36–2.15), and MCI (OR 1.61, 95% CI: 1.28–2.03). In contrast, these dementia types were associated with lower odds of receiving OT. Older age was negatively associated with SLT use, whereas no consistent age-related pattern was observed for OT utilisation.

**Conclusion:**

Our findings reveal low utilisation of SLT and OT, highlighting significant gaps in allied health service provision for people with dementia or MCI. These results underscore the need for improved referral pathways and targeted strategies to better integrate allied health professionals into routine dementia care.

**Trial registration:**

The study was not registered.

**Supplementary Information:**

The online version contains supplementary material available at 10.1186/s12913-025-13149-y.

## Introduction

Dementia is one of the leading causes of disability and care dependency among older adults worldwide, currently affecting an estimated 55 million people [1], including approximately 1.8 million in Germany [2]. The syndrome is characterised by a progressive decline in cognitive functions and daily living abilities, resulting in impairments in orientation, communication, and episodic memory. In addition to age, modifiable factors such as cardiovascular health, lifestyle behaviours, and psychosocial conditions increase the risk of developing dementia [1, 3].

Alzheimer’s disease (AD) is the most common form of dementia, accounting for 60–80% of all cases, followed by vascular dementia (5–10%) and less common types such as dementia with Lewy bodies (5%) and frontotemporal dementia (3%) [4]. Clinically, dementia is staged into three phases (mild, moderate and severe), and is often preceded by mild cognitive impairment (MCI), a multifactorial condition characterised by measurable cognitive decline that does not interfere with independent daily functioning [5]. In Germany, an estimated 3.7 million people are affected by MCI [6]. As a major risk syndrome with a threefold increased likelihood of progressing to dementia [7], MCI represents a critical window for early detection and timely intervention to decelerate or prevent further cognitive decline [8].

While pharmacological treatments (e.g., rivastigmine, galantamine) offer limited symptomatic relief [5], newer promising approaches such as lecanemab aim to slow disease progression by targeting neuropathological processes in the early stages of dementia and in individuals with MCI [9]. Besides, allied health professionals (AHPs), including speech-language therapists (SLTs) and occupational therapists (OTs), provide essential non-pharmacological interventions to support cognitive function, daily living abilities, and quality of life [10, 11]. These interventions target both cognitive and functional impairment and are delivered in individual or group-based formats. Collectively, they are referred to as cognitive interventions and may also be provided by neuropsychologists, nurses, or social workers [5]. However, SLTs and OTs are the main providers of reimbursable cognitive therapies in German outpatient care [12], delivering the majority of services across more than 20,000 therapy practices [13]. By contrast, only around 200 neuropsychologists currently work in an outpatient capacity [14]. Nurses and social workers typically provide cognitive interventions as part of in-house care and support services, which are not widely available and cannot be directly prescribed. SLTs and OTs are thus central to the structured and reimbursed provision of cognitive interventions.

Cognitive interventions include cognitive stimulation therapy (CST), cognitive training (CT), and cognitive rehabilitation. CST is a group-based program focusing on memory, attention, and communication. CT targets specific cognitive functions through structured exercises, while rehabilitation typically involves environmental modifications and caregiver training [5]. In recent years, the role of SLTs in cognitive dementia care has received growing attention, as progressive language and communication impairments occur in all neurodegenerative dementia types [15, 16] and are often under-recognised [15]. Symptoms such as word-finding difficulties or problems following complex conversations are among the earliest symptoms of MCI and early-stage AD [15, 17]. While often overlooked in typical dementia subtypes [15], language impairments are the core diagnostic criterion in rarer forms such as primary progressive aphasia (PPA) [5]. Overall, communication impairments are considered one of the most significant unmet needs in people with dementia (PWD) [15]. SLT specifically addresses language as a cognitive function and supports communicative participation. SLT interventions may include cognitive training like word-retrieval exercises or naming therapy, and caregiver-based communication strategies [18]. These interventions are individually tailored in close consultation with patients, families, and interdisciplinary teams [19]. In addition, SLTs provide dysphagia therapy, which becomes relevant in up to 85% of individuals in later stages of dementia [20, 21].

OT typically addresses broader cognitive functioning and daily-life performance, as well as neuropsychiatric symptoms and care partner support in PWD/MCI [22]. Depression commonly occurs in association with MCI and dementia, and behavioural and psychological symptoms of dementia are frequent across the disease continuum [5]. Various approaches are used, including CST, reminiscence therapy (e.g. biographical memory work), and neuropsychological memory training techniques such as spaced retrieval or errorless learning [23].

Meta-analyses indicate that cognitive interventions may slow cognitive decline [8, 24] and reduce depressive symptoms in individuals with MCI [25]. Evidence from controlled trials suggests positive effects on cognition, communication and quality of life in people with mild to moderate dementia [18, 26–29], and potential benefits for communicative participation and quality of life have also been reported in moderate to severe stages [18]. CST is among the most extensively studied approaches and is considered cost-effective compared to standard care [30]. SLT-based partner-communication training has been shown to reduce caregiver burden [31, 32], which is a major factor contributing to the institutionalisation of PWD [10].

Despite this growing body of evidence, little is known about the real-world implementation of SLT and OT in dementia care. Although current guidelines recommend cognitive interventions, particularly in early stages [5], few studies have investigated how SLT and OT are used in practice. Available studies are based on small and incomplete samples [33–35] and do not differentiate between treatment indications. As a result, it remains unclear whether the treatments were prescribed primarily to address dementia-related cognitive impairment or other comorbidities, such as dysphagia [33–35]. Given the increasing complexity of dementia care, it is essential to use all available resources effectively, particularly in order to prevent the transition from MCI to dementia and to slow disease progression where possible. Real-world care data offer an opportunity to address this evidence gap by providing insights into actual service use, prescription patterns, and influencing factors [36].

This study therefore aims to:

I. describe the use of SLT and OT by PWD/MCI in Germany within the first two years after diagnosis and
II. examine socio-demographic and clinical factors associated with the use of SLT and OT.

## Methods

### Study Design

This study was a retrospective longitudinal study using routinely collected claims-based data administrated by the Institute of Applied Health Research Berlin (InGef) research database. The dataset covered the period from 1 January 2017 to 31 December 2022. To examine the use of SLT and OT, all incident cases with an initial diagnosis of dementia or MCI between 1 January 2017 and 31 December 2020 (inclusion period) were identified and followed for an individual observation period of two years from the date of diagnosis. The study was descriptive; thus, no hypotheses were pre-specified. It was designed, conducted, and analysed in accordance with guidelines and recommendations for good practice in secondary data analysis [37]. Reporting followed the RECORD statement for observational studies using routinely collected health data [38] (see Supplementary Material, Table S1).

### Data Source

The InGef database [39] is an anonymised health claims database containing longitudinal data on approximately 9 million individuals in Germany. These individuals are insured with one of over 60 statutory health insurance (SHI) funds, primarily company or guild-based insurance funds. Claims data can be traced back over a period of six years. For this analysis, an age- and sex representative sample of approximately 5 million individuals from 2017 to 2022 was selected. The database included socio-demographic data such as age, sex and region of residence, as well as diagnoses from outpatient and inpatient sectors. It also contained information on healthcare services and procedures, reimbursed non-pharmacological therapies, medical aids, and pharmaceuticals. Each diagnosis or treatment included information on the profession of the treating physician. Diagnoses were coded according to the German Modification of the international Classification of Diseases 10th Revision (ICD-10-GM). Non-pharmacological therapies were documented by corresponding therapeutic appliance item numbers according to the specifications of the German Catalogue of Therapeutic Appliances. All patient-level data were de-identified in compliance with German Data Protection Regulation (§ 67 Abs. 2 SGB X i.V.m. Art. 4 Nr. 1 DSGVO), and the use of the database for health services research was fully compliant with German federal law. Subsequently, ethical approval was not required for this study.

### Population

Adult patients aged 18 years and older were included in the study population if they were continuously insured with one of the SHI organisations included in the InGef database during the observation period or until death. This criterion ensured the availability of complete claims data, independent of healthcare utilisation frequency. Analyses of patient characteristics and therapy utilisation were conducted in the subpopulation with a newly confirmed diagnosis of dementia or MCI. Only psychiatric diagnoses from Chapter V of the ICD-10-GM indicating clinically manifest dementia or MCI were considered: F00.-* (Dementia in AD), F01.-* (vascular dementia), F02.-* (dementia in other diseases classified elsewhere), F03 (unspecified dementia), and F06.7 (MCI). The code F02 refers to dementia as a consequence of other diseases classified elsewhere in the ICD-10-GM system, such as Parkinson’s disease or Pick’s disease. The code unspecified dementia is used when clinical criteria for dementia are met, but no specific etiology can yet be established, unlike F00–F02, which reflect defined underlying causes.

Diagnoses had to be documented either in outpatient care or as a main or secondary hospital discharge diagnosis. An outpatient diagnosis was considered confirmed if it was documented in two separate quarters in the index year (year of the initial diagnosis) or by two different physicians in the same quarter. To determine the incidence, a disease-free period of one year prior to the initial diagnosis was required. Incident cases from 2017 onwards were identified by using 2016 as a validation period. Patients firstly diagnosed with MCI or any form of dementia between 1 January 2017 and 31 December 2020 who could be followed for two years or until death were included (patient-individual period). An incident case entered the study population at the beginning of the quarter in which the dementia/MCI diagnosis was first documented. Study exit was defined as the end of the observation period or death. There were no exclusion criteria.

### Measures

The primary outcome was the use of SLT and OT by PWD/MCI. According to the German Catalogue of Therapeutic Services, these services must be prescribed by physicians to be reimbursed by the health insurers related to different indication codes. These codes also indicate the medical reason for the treatment. However, these codes often cover a wide range of conditions and do not provide information about the exact underlying disease. For this reason, the study linked the ICD diagnosis data to the use of SLT and OT on an individual basis.

The first step of the analysis assessed the overall proportion of patients who received SLT or OT, regardless of the indication. The second step examined whether these patients were being specifically treated concerning their diagnosis of dementia/MCI. In the case of SLT, a distinction was made between therapy for cognitive communication impairments (indication code: SP5) and therapy for dysphagia (indication code: SC), as these are dementia-related interventions. As both indications can be prescribed for different neurological conditions, physicians have the option to voluntarily document up to four ICD codes relevant to treatment on the prescription. These were also checked to see if a dementia/MCI diagnosis was recorded (dementia-specification of the prescription). OT already has a dementia-specific code (PS5 until 12/2020, PS4 since 1/2021) that allowed billing for cognitive training or psycho-functional treatment [12]. In the following, we refer to OT prescriptions using this code as “dementia- specific OT”. Different services may be ordered per prescription (e.g. 30 min of individual SLT or OT cognitive training). SLT prescriptions typically cover 10–20 therapy sessions, and OT prescription 10 sessions. A home visit could be added to any prescription. Group therapy was available for both SLT, focusing on communication, and for OT, focusing on dementia- specific interventions [12]. Definitions and codes of all variables used are provided in the Supplementary Material (Table S2). Patients who received at least one prescription during the follow-up period were considered to be patients who received treatment. Additionally, the number of prescriptions per patient and the number of therapy sessions billed were quantified. For each prescription, the professional subject of the prescribing physician was identified. The regularity of therapy provision was also assessed. Regular care was defined as having at least one prescription in at least three of the eight quarters of follow-up. With regard to socio-demographic factors, age and sex were examined. Age was calculated in the quarter of the initial diagnosis and patients were grouped into age categories ranging from: 18–64, 65–69, 70–74, 75–79, 80–84, 85–89, ≥ 90 years. Clinical variables included the type of dementia and the clinical discipline of the prescribing physician. If multiple diagnoses were recorded in the index quarter, the first-listed diagnosis was used. All experts relevant for the treatment of dementia/MCI were included.

### Analysis

To assess the utilisation of SLT and OT during the two-year patient-specific follow-up, absolute and relative frequencies of therapy use were calculated for each included indication. The number of prescriptions and billed therapy sessions were summarised using the mean, median, and interquartile range (IQR).

To examine associations between patient characteristics and therapy utilisation, multivariate logistic regression models were applied separately for each therapy indication (SLT: communication impairment, SLT: dysphagia, OT). The dependent variable in each model was binary, indicating whether the patient received at least one prescription for the respective therapy during the follow-up period (yes/no). Predictor variables included age (categorised as: 18–64, 65–69, 70–74, 75–79, 80–84, 85–89, and ≥ 90 years), sex (male/female), and dementia diagnosis (AD, vascular dementia, dementia classified elsewhere, unspecified dementia, and MCI). The regression models were calculated using a 95% confidence level (α = 0.05). Results are reported as odds ratios (ORs) with corresponding 95% confidence intervals (CI) and p-values. All analyses were exploratory and performed by InGef staff using the statistical software R [40], version 4.0.2 (available at: https://www.r-project.org/).

## Results

### Population size

**Fig. 1:**
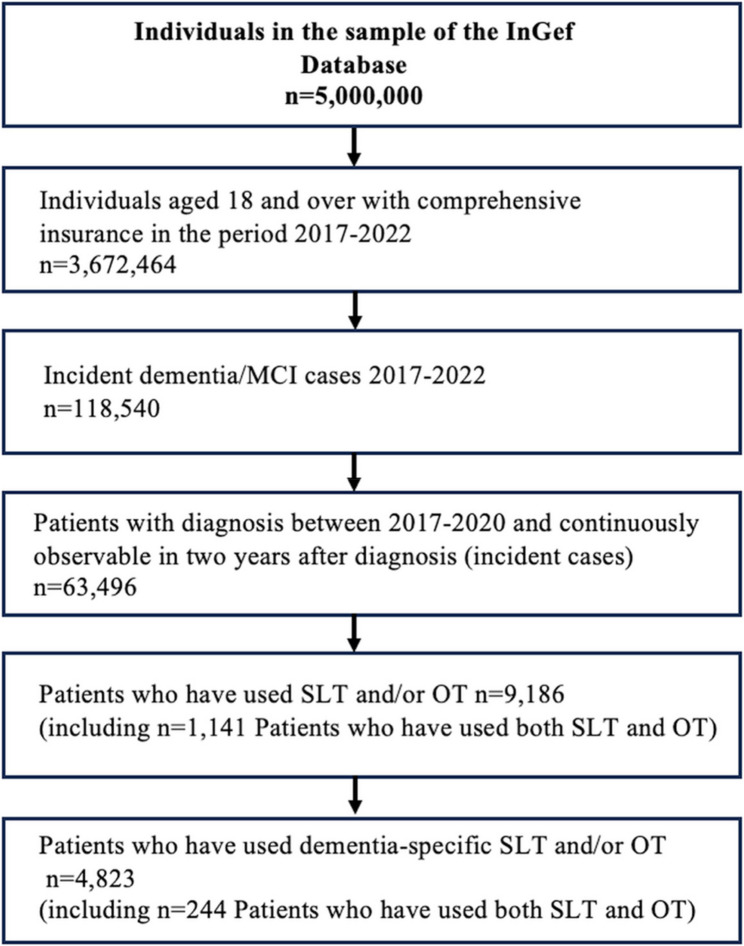
Data flow of sample selection. Annotations: Flowchart of sample selection from the InGef database, n=63,496 individuals with dementia or MCI. Abbreviations: InGef = Institute for Applied Health Research Berlin; MCI = mild cognitive impairment; OT = occupational therapy; SLT = speech-language therapy

Out of the sample of 5 million insured persons in the InGef database, 3,672,464 were aged 18 years or older and continuously insured in the period 2017–2022 or until death. Fig. 1 shows the data flow.

### Study population

A total of 63,496 individuals were included in the study cohort, including 51,941 PWD (81.8%) and 11,555 persons with MCI (18.2%). During individual follow-up, 18,018 participants (28.4%) died. The median age at first diagnosis was 82 years (IQR: [10]) for PWD and 76 years (IQR: [14]) for those with MCI. The majority of patients were female (58.0%). Most PWD were diagnosed with unspecified dementia (45.4%), followed by vascular dementia (17.9%) and AD (15.5%). Table 1 summarises the descriptive characteristics of the study population.

### Utilisation of SLT and OT

Overall, 4.2% (*n* = 2,661) of the study population received SLT, while 10.3% (*n* = 6,525) received OT, indicating that OT was prescribed more than twice as often as SLT. A total of 1.8% (*n* = 1,141) received both therapies. In sum, 12.7% (*n* = 8,045) received at least one of these servicesAmong those receiving OT (*n* = 6,525), only 42.8% were treated specifically for dementia (*n* = 2,795). The remaining PWD/MCI underwent therapy for other indications, e.g. spinal or central nervous system disorders. 

Regarding SLT, 76.2% (*n* = 2,028) of all patients who received therapy (*n* = 2661) were treated for dementia-related reasons. These were proportionally distributed between the two primary indications: 38.6% for SLT communication and 37.6% for SLT dysphagia disorders (see Table 1). The remaining patients who received SLT were treated for unrelated indications, most commonly motor speech or voice disorders.

**Table 1:** Characteristics of the study population (*N* = 63,496)

Variable	Individuals (*n* = 63,496)	%
*Sex*		
Male	26,671	42.0
Female	36,825	58.0
* Age (in groups)*		
18–64	4,118	6.5
65–69	2,878	4.5
70–74	6,318	10.0
75–79	12,370	19.5
80–84	16,494	26.0
85–89	12,632	19.9
≥90	8,686	13.7
* Dementia type*		
AD	9,816	15.5
Vascular dementia	11,369	17.9
Dementia classified elsewhere	1,956	3.1
Unspecified Dementia	28,800	45.4
MCI	11,555	18.2
*Therapy use general*		
SLT	2,661	4.2
OT	6,525	10.3
Both SLT and OT	1,141	1.8
* Therapy use according to indication*		
SLT communication	1,027	1.6
SLT dysphagia	1,001	1.6
Dementia-specific OT	2,795	4.4
Dementia-specific OT and SLT	244	0.4
Dementia-specific OT and SLT communication	106	0.2

Annotations: Percentages refer to the total study population

*Abbreviations*: *AD* Alzheimer’s disease, *MCI* mild cognitive impairment, *SLT* speech-language therapy, *OT* occupational therapy

**Fig. 2:**
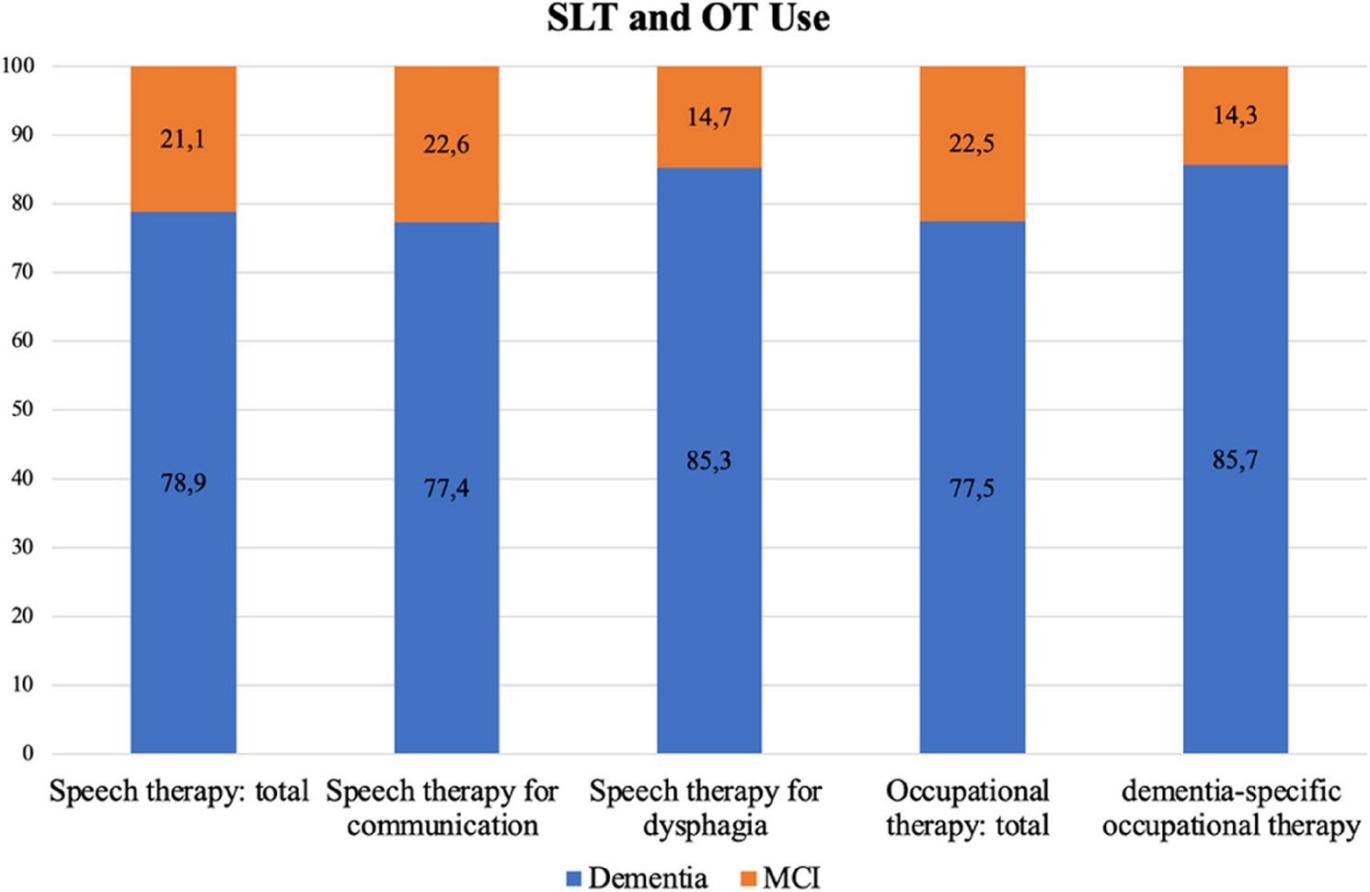
Therapy utilisation by indication, distributed by Dementia or MCI. Annotations: Distribution of all patients receiving SLT or OT (*n* = 8, 045) by diagnosis (dementia or MCI) and therapy indication. Abbreviations: MCI, mild cognitive impairment; OT, occupational therapy; SLT, speech-language therap

As shown in Fig. 2, the proportion of individuals with MCI among those receiving SLT or OT ranged between 14.3% and 22.6%, depending on the indication.

### Prescribing patterns

During the two-year observation period, patients who received dementia-related SLT had a median of three prescriptions for communication therapy (IQR: [4]) and two prescriptions for dysphagia therapy (IQR: [3]). An explicit reference to a dementia or MCI diagnosis was documented on only a small proportion of these prescriptions (8.2% communication prescriptions, 2.4% dysphagia prescriptions).

For dementia-specific OT, the median number of prescriptions was two (IQR: [4]). Approximately half of the patients received therapy on a regular basis (SLT for communication: 55.3%, dementia-specific OT: 45.2%).

In SLT, substantial discrepancies were observed between the mean and median numbers of prescriptions and reimbursed therapy sessions, which may be due to extreme outliers.

The majority of SLT for communication impairment (85.6%) and dysphagia (92.3%) was documented as home-based therapy sessions. A high proportion of OT prescriptions for dementia also included home visits (50.9%). Further details are presented in Table 2.

**Table 2:** Non-pharmacological therapy utilisation rates among the study population (*N* = 63,496)

Dementia/MCI patients, *n* (%)	63,496 (100)							
	n	(%)	Prescription per patient (mean, median, IQR)			Therapy sessions (mean, median, IQR)		
SLT total	2,661	4.2	3.6	2	4	196.5	61	159
SLT: communication	1,027	1.6	3.6	3	4	193.1	60	150
Prescription with home visit	879	1.4	4.3	2	4	176.8	30	140
Regular prescriptions Prescription with dementia/MCI code	568 84	0.9 0.1	5.3 2.1	5 2	4 1	293.1 82.3	107 40	229 68
SLT: dysphagia	1,001	1.6	3.1	2	3	187.5	48	149
Prescription with home visit	924	1.5	3.1	2	3	170.0	33	121
Regular prescriptions Prescription with dementia/MCI code	391 24	0.6 0.04	5.6 1.7	5 1	4 1	369.2 63.7	162 42	314 46
OT total	6,525	10.3	4.0	3	5	61.4	31	63
Dementia-specific OT	2,795	4.4	3.5	2	4	58.5	34	63
Prescription with home visit	1,422	2.2	3.7	3	4	44.9	30	48
Regular prescription	1,264	2.0	5.9	5	3	101.3	80	70

Annotations: Patients with at least one prescription. Regular prescription was defined as having received at least one prescription in at least three out of the eight quarters of follow-up

*Abbreviations*: *IQR* interquartile range, *MCI* mild cognitive impairment, *OT* occupational therapy, *SLT* speech-language therapy

Most patients with SLT for communication impairments received a prescription for 45 min therapy (77.8% of all prescriptions issued), followed by 30 min (15.3%). Group therapy was rarely prescribed (0.7%). A similar pattern was observed in dysphagia therapy, where 45 min-sessions were most commonly prescribed (69.8%), followed by 30-minute sessions (23.4%).

In dementia-specific OT, cognitive training was the most frequently prescribed intervention (55.7%), closely followed by psycho-functional treatment (42.3%). Group therapy accounted for only 1.9% of all prescribed services.

Neurologists accounted for the largest proportion of SLT prescriptions for communication impairments (34.6%) and dementia-specific OT prescriptions (44.2%), followed by general practitioners (SLT: 33.2%, OT: 20.8%) and internists with a specialisation in geriatric care (SLT: 19.1%, OT: 10.2%). In contrast, dysphagia therapy was most frequently prescribed by general practitioners (40.5%), followed by neurologists (22.0%) and geriatric internists (21.0%).

### Association of socio-demographic and clinical factors with the utilisation of SLT and OT services

Multivariate logistic regression models were conducted separately for each indication to examine factors associated with the use of SLT (for communication impairment and dysphagia) and dementia-specific OT.

For SLT, both sex and type of dementia were significant predictors. Men were more likely than women to receive SLT for both communication (OR1.24, 95% CI 1.09–1.40) and dysphagia (OR 1.53, 95% CI 1.35–1.73). Regarding dementia type, individuals diagnosed with dementia classified elsewhere had substantially higher odds of receiving SLT– more than three times higher for communication therapy (OR 3.34, 95% CI 2.46–4.53) and over four times higher for dysphagia therapy (OR 4.12, 95% CI 3.04–5.55) compared to patients with AD. Vascular dementia was also significantly associated with higher SLT use (communication: OR 1.71, 95% CI 1.36–2.15; dysphagia: OR 1.86, 95% CI 1.47–2.36). MCI was associated with higher SLT use only for communication (OR 1.61, 95% CI 1.28–2.03), but not for dysphagia. This indicated that certain dementia types were significantly more likely to receive SLT compared to AD.

Age showed a consistent negative association: compared with those aged 18–64, all older groups had progressively lower odds of receiving SLT. Detailed trends are shown in Figures S1 and S2 (Supplementary Material).

In contrast, the use of OT followed a different pattern. Higher odds were observed in the 65–69 (OR 1.46, 95% CI 1.18–1.81) and 75–79 (OR 1.34, 95% CI 1.14–1.60) age groups, but there was no consistent trend across age groups. In particular, people aged ≥ 85 years were less likely to receive OT than younger groups. In contrast to SLT, there were no significant sex differences in OT use. Dementia type again played a role: vascular dementia (OR 0.39, 95% CI 0.34–0.44), dementia classified elsewhere (OR 0.26, 95% CI 0.18–0.35), unspecified dementia and MCI were all associated with significantly lower odds of OT use compared to AD. Complete model results are presented in Table 3.

**Table 3:** Multivariable associations between SLT and OT utilisation and socio-demographic, and clinical characteristics (*N* = 63,496)

Variables	Utilisation– logistic regression											
	SLT: communication				SLT: dysphagia				OT			
	OR	95% CI		p value	OR	95% CI		p-value	OR	95% CI		p-value
Female	1	0.885	1.129	1	1	0.878	1.139	1	1	0.932	1.073	1
Male	1.235	1.089	1.400	< 0.001	1.527	1.345	1.734	< 0.001	0.937	0.867	1.013	0.100
18–64 years	1	0.811	1.232	1	1	0.750	1.333	1	1	0.806	1.240	1
65–69 years	0.694	0.535	0.895	0.004	1.169	0.861	1.584	0.325	1.460	1.176	1.813	< 0.001
70–74 years	0.476	0.380	0.595	< 0.001	0.672	0.505	0.893	0.005	1.174	0.972	1.421	0.094
75–79 years	0.337	0.275	0.414	< 0.001	0.594	0.463	0.767	< 0.001	1.344	1.138	1.596	< 0.001
89 − 84 years	0.254	0.207	0.312	< 0.001	0.628	0.496	0.800	< 0.001	1.095	0.927	1.297	0.288
85–89 years	0.212	0.168	0.267	< 0.001	0.537	0.417	0.695	< 0.001	0.687	0.572	0.826	< 0.001
≥ 90 years	0.112	0.080	0.154	< 0.001	0.445	0.332	0.594	< 0.001	0.465	0.376	0.576	< 0.001
AD	1	0.767	1.130	1	1	0.757	1.320	1	1	0.904	1.106	1
Vascular dementia	1.706	1.359	2.151	< 0.001	1.858	1.470	2.360	<0.001	0.389	0.344	0.440	< 0.001
Classified elsewhere	3.343	2.457	4.525	< 0.001	4.117	3.043	5.553	<0.001	0.255	0.184	0.345	< 0.001
Unspecified dementia	0.973	0.787	1.209	0.789	1.243	1.003	1.552	0.046	0.370	0.335	0.405	< 0.001
MCI	1.607	1.280	2.027	< 0.001	1.105	0.855	1.433	0.447	0.355	0.313	0.041	< 0.001

Annotations: Reference categories: AD for dementia type, female for sex, and 18–64 years for age group. ORs > 1 indicate higher likelihood of therapy use compared to the reference group

*Abbreviations*: *AD* Alzheimer’s disease, *CI* confidence interval, *MCI* mild cognitive impairment, *OR* odds Ratio, *OT* occupational therapy, *SLT* speech-language therapy

## Discussion

### Summary of findings

This study analysed the utilisation and prescription patterns of SLT and OT in PWD/MCI in Germany, using data from the InGef database (*n* = 63,496). Within two years of diagnosis, only 1.6% of patients received SLT for communication impairments, and 4.4% received dementia- specific OT, indicating an overall low uptake of cognitive interventions for dementia- and MCI-related symptoms. Approximately half of the patients received therapy on a regular basis (SLT: 55.3%, OT: 45.2%), and services were mainly provided in home-based settings (SLT: 85.6%, OT: 50.9%).

Multivariable logistic regression showed that male sex and certain dementia types (e.g. vascular dementia and dementia classified elsewhere) were associated with higher SLT use, whereas increasing age was associated with lower utilisation. In contrast, OT use was lower among individuals with non-AD and MCI, and showed no consistent association with age. Overall, the results suggest disparities in therapy access depending on male sex, younger age and type of dementia.

### Discussion of findings

To our knowledge, this is the first comprehensive study on SLT and OT use among PWD/MCI in Germany, based on a large, representative dataset. Previous research has primarily focused on PWD and relied on survey-based methods, often without indication-specific analyses. For example, Wübbeler et al. [34] examined the use of physiotherapy and OT by *n* = 560 caregivers of PWD in dementia networks throughout Germany. The results showed a comparatively higher use of OT (15.5%), but did not differentiate by indication. Kohler et al. 2014 [35] reported utilisation rates more comparable to our findings: only 3.1% of study participants (*n* = 203 PWD) received OT and 1.9% received SLT. A meta-analysis by Michalowsky et al. [33] (*n* = 103,980) found an average of 6.9 therapy sessions per year for PWD living at home, compared with 10.2 sessions in residential care. In contrast, our study revealed substantially higher therapy volumes: a median of 60 sessions per patient for SLT and 34 for OT within two years of diagnosis. However, the data reported by Michalowsky et al. [33] are only partially comparable due to differences in the combination of therapy services, calculation methods and time periods analysed [33].

It should be noted that the study period also includes the COVID-19 pandemic and the associated lockdowns in Germany (March–May 2020 and November 2020–May 2021) [41]. Routine data from Germany’s largest statutory health insurer showed that the overall utilisation of SLT and OT decreased in 2020 by 7.5% and 6.2%, respectively, compared to 2019. However, by the end of 2020, utilisation had largely returned to pre-pandemic levels [42]. Given this relatively short disruption and the two-year observation period per individual, the pandemic is unlikely to have had a substantial influence on the overall findings.

In addition to external factors such as the pandemic, therapy utilisation must also be viewed in the clinical context of disease progression in dementia. This perspective is lacking in the cited studies. According to observational studies, the average disease duration from diagnosis to death is 4.3–5.1 years (IQR: 2.3-8.0) [43]. In clinical practice, continuous therapy over the entire course of the disease is rarely implemented, as the intensity of treatment is typically prioritised during phases of higher expected benefit [44]. For this reason, our study focused on the first two years following diagnosis. It should be noted, however, that the coding of a formal diagnosis does not necessarily reflect the actual onset of dementia. Dementia is often diagnosed at an advanced stage. According to a survey of *n* = 276 PWD in Germany, the median time between first symptoms and formal diagnosis was 16 months [45]. With that in mind, the diagnosis of MCI represents a timely opportunity for early intervention and monitoring to achieve a delay in disease progression [46].

In our study, one in five patients who received cognitive interventions from an SLT and one in seven patients who received cognitive interventions from an OT had a diagnosis of MCI. This indicates that the treatment of individuals with MCI is already part of current clinical practice. However, this level of care provision should be further developed and standardised to ensure earlier and more consistent support for this patient group. The majority of people with MCI in our sample received SLT for communication impairments, and logistic regression confirmed a positive association between MCI and SLT utilisation. This is particularly notable, as language and communication disorders are strong predictors of the progression from MCI to dementia [17]. SLTs can therefore play a special role in dementia care, taking on important tasks such as monitoring and early detection [46].

Overall, OT was prescribed more frequently than SLT. This aligns with the well-established role of OT in dementia care, which is supported by reimbursement policies and its inclusion in both the German S3 guideline on dementia [5] and the national dementia strategy [47]. In contrast, SLT is mentioned less frequently and remains underrepresented in interdisciplinary research and care models [26]. Nonetheless, the international discourse increasingly recognises the relevance of SLT in dementia care [48]. Leading professional associations such as the American Speech-Language-Hearing Association (ASHA) [19] and the Royal College of Speech Therapists (RCSLT) [49] have published position papers outlining the profession’s scope and responsibilities within interdisciplinary dementia care teams. According to ASHA´s annual survey, communication disorders in dementia now represent the second largest treatment group in the adult sector [50]. This level of visibility and professional recognition has yet to be established in the German context.

Our findings also indicate that SLTs were only marginally involved in the treatment of dysphagia among PWD. Given that the German S3 guideline explicitly mandates SLT involvement in swallowing therapy [5], a higher utilisation rate would have been expected. One possible explanation is that dysphagia typically occurs in later stages of dementia and may not have been captured during the two-year follow-up period. Additionally, it is likely that the small number of patients with MCI who received dysphagia therapy were treated for comorbid conditions such as stroke or Parkinson’s disease, rather than for dementia-related symptoms.

In general, SLT utilisation must be interpreted in the context of the underlying data structure. Unlike OT, it is not possible to reliably determine from the available data whether SLT was prescribed for dementia/MCI or for other comorbid conditions. Since treatment-relevant diagnoses are not required on prescriptions, our findings may overestimate dementia-specific SLT use. This interpretation is further supported by the logistic regression results, which showed significantly higher odds of receiving SLT among patients with vascular dementia and dementia associated with other neurological diseases compared to those with AD. This suggests that SLT was often prescribed to address comorbidities rather than dementia symptoms. Its established use in conditions such as stroke and Parkinson’s disease may explain the more frequent application in these dementia subtypes. In contrast, OT was less frequently prescribed for non-AD patients, possibly reflecting its stronger emphasis in clinical research and treatment guidelines for AD compared to other dementia types [22].

In addition to clinical characteristics, individual health behaviours and attitudes may influence therapy utilisation. These factors can be systematised using Andersen’s behavioural model of health services utilisation, which distinguishes between three core components: predisposing characteristics, enabling resources, and individual need. Predisposing factors include demographic and social characteristics [51]. Our findings point to disparities in predisposing characteristics: Men were significantly more likely to receive SLT. This is particularly noteworthy given that women are not only more frequently affected by dementia, but also assume the majority of informal caregiving responsibilities, providing up to 70% of care hours for PWD [1]. These findings may reflect sex-based differences in referral patterns or help-seeking behaviour. It is also plausible that women, as primary caregivers, are more actively involved in organising healthcare and therapy services—potentially leading to higher therapy uptake among male patients [52]. In addition, biological and cognitive factors may contribute to these disparities. Research suggests that women with early-stage AD or MCI tend to outperform men on verbal memory tasks, potentially delaying the recognition of language-related deficits and subsequent referral to SLT [53]. Similarly, older people were consistently less likely to receive SLT, even though communication and swallowing difficulties typically increase as dementia progresses. In contrast, our findings did not show a consistent age-related trend in OT use, although previous studies suggest that older age and a higher number of comorbidities are associated with increased OT utilisation [34].

Enabling or impeding factors refer to individual and structural resources that influence access to care, including educational level, socioeconomic status and physical accessibility [51]. Among PWD, restricted mobility and the need for support from care partners are well-documented barriers to therapy use [10]. Notably, our results indicate that both SLT and OT were more frequently provided through home visits, suggesting that increased awareness of this care modality may help to overcome some of these structural barriers.

The third component—individual need—includes perceived and professionally evaluated health needs [51]. Among PWD/MCI, limited awareness of available services may restrict therapy utilisation [10]. Moreover, individual-level barriers such as low health literacy, entrenched health behaviours, and psychological factors, including comorbid affective disorders such as depression, can further compromise the perceived relevance and uptake of therapeutic services [54].

Finally, Andersen’s model also incorporates contextual factors, including those on the provider side [51]. In Germany, usually PWD/MCI firstly get in touch with general practitioners, as they typically make the initial diagnosis and coordinate referrals to AHP-led service [55]. Existing research [10, 56, 57] highlights several practical and attitudinal barriers that contribute to explain the restricted use of these interventions. One commonly assumed explanation is the perception of financial restrictions. While AHP services are generally subject to budgetary limits in Germany, dementia is classified as a special prescription need, which exempts these therapies from standard cost-effectiveness evaluations [58]. This suggests that formal structural restrictions may not represent the main barrier. Qualitative findings by Wangler et al. (2020) [56] comprising *n* = 41 general practitioners, display additional practice-level barriers, including time and resource constraints, as well as scepticism regarding the benefits of non-pharmacological therapies [56]. This form of therapeutic nihilism is also reflected in international surveys, with both medical professionals and AHPs expressing concerns about whether PWD can meaningfully engage in or benefit from cognitive interventions [10, 57]. Such attitudes may contribute to under-diagnosis, low referral rates and missed opportunities for supportive care [57].

These perceptions may stem from a lack of knowledge and training about effective cognitive interventions for dementia-related cognitive decline. Targeted education is needed to address this gap, not only for prescribers but also for AHPs involved in dementia care [10, 55]. Therapists themselves often report uncertainty, for instance, in setting realistic and measurable therapy goals for PWD/MCI [57]. This is particularly evident in the area of SLT [48]. In Germany, dementia-specific interventions are not a standard part of SLT training programmes, whereas OT education typically includes relevant competencies [59, 60]. This indicates the need for improved education, training programmes and clear guidelines to support therapists in practice. In addition to training, strengthening collaboration between OTs, SLTs, and neuropsychologists within an interdisciplinary cognitive rehabilitation framework is essential. Clearer role delineation, joint diagnostic and therapeutic planning could support more effective teamwork and improve care [26].

Another barrier to therapy access is a general shortage of qualified professionals available to deliver SLT and OT services in Germany [61]. To address this, more resource-efficient formats should be considered. One promising approach is group therapy, which allows a single therapist to treat several patients simultaneously. Despite its potential, group-based cognitive interventions such as CST remain underutilised in practice. This finding highlights the need for further research into barriers to implementation and the development of strategies to increase uptake. Additional research is also needed to evaluate the optimal duration, intensity, and cost-effectiveness of cognitive interventions for PWD/MCI in real-world care settings. Given that our findings indicate a median of 34–60 sessions per patient over two years, this may serve as a feasible benchmark for routine care. Cognitive interventions that also aim to reduce carer burden - as well as communication or educational training in a group setting - offer further potential [62]. Finally, it is equally important to ensure that both health professionals and PWD/MCI have access to explicit and comprehensive information about available therapeutic services.

## Limitations

The study is notable for its large, unselected, nationwide sample, which allows a robust estimate of the use of SLT and OT among PWD/MCI in Germany. Although only a limited number of SHI funds are included in the InGef database, previous analyses of the external validity show a good correspondence between the data set and the German population [39]. The use of routine data reduces the risk of bias due to non-response, recall or selection bias. Moreover, secondary data allow the inclusion of multimorbid and institutionalised individuals, who are often excluded from primary surveys. These characteristics make routine data particularly valuable for dementia-related health services research [63].

Nevertheless, the findings must be interpreted with caution, as claims data are primarily collected for billing purposes. They reflect diagnostic and prescribing behaviour rather than actual disease prevalence or clinical needs and are subject to limitations regarding data quality and validity. This is particularly relevant in the context of diagnosing MCI, dementia, and its subtypes, as dementia diagnoses in claims data are not standardised and do not correspond to prospective clinical assessments. In outpatient care, misclassifications and the frequent use of unspecific diagnoses are common, as also reflected in our analysis. Furthermore, the diagnosis of MCI is used heterogeneously in clinical practice and may represent a wide range of underlying conditions [5].

Clinical parameters such as disease severity, functional status, or exact duration of diagnosis could not be assessed. Additionally, it was not possible to determine whether patients who received SLT or OT had previously undergone similar treatment prior to their dementia or MCI diagnosis. Therefore, the entire analysis must be interpreted in the context of possible confounding by other diseases. This applies in particular to affective disorders [5], which may confound therapy utilisation patterns by influencing diagnosis, patient engagement, or referral behaviour. For a more accurate analysis, co-morbidities should have been included.

Another key limitation relates to the specificity of treatment indications. Due to structural limitations in the German billing system, it was not always possible to determine whether SLT was prescribed for dementia-related symptoms or for other neurological conditions. This ambiguity may have led to an overestimation of dementia-specific SLT use. Furthermore, the data solely captures treatments billed to the SHIs, not those services that were excluded from reimbursement. Prescriptions from self-pay patients could not be included. Patients with private health insurance were excluded from the dataset, which may limit generalisability of the findings to the entire population.

## Conclusion

SLT and OT are valuable resources in non-pharmacological dementia care. This study is the first comprehensive analysis of SLT and OT utilisation among PWD/MCI in Germany using nationwide health claims data. Our findings show that these services remain underutilised in routine care. The low uptake of SLT and OT, particularly among people with MCI, suggests missed opportunities for early intervention. The disparities observed in utilisation according to sex, age, and dementia type may reflect barriers to access and referral. Further research is needed to explore these disparities and develop targeted strategies to improve care pathways and integrate SLT and OT more effectively into routine dementia care.

## Supplementary Information

Supplementary Material 1

## Data Availability

The data used in this study cannot be made available in the manuscript, the supplemental files, or in a public repository due to German data protection laws (Bundesdatenschutzgesetz). To facilitate the replication of results, anonymized data used for this study are stored on a secure drive at the InGef - Institute for Applied Health Research Berlin GmbH. Access to the raw data used in this study can only be provided to external parties under the conditions of a cooperation contract and can be accessed upon request, after written approval (info@ingef.de), if required.

## Abbreviations

AD: Alzheimer’s Disease/ Alzheimer’s Dementia
ASHA: American Speech-Language-Hearing Association
CST: Cognitive stimulation therapy
DSGVO: German Data Protection Regulation (Datenschutz-Grundverordnung)
ICD: International classification of Diseases
InGef: Institute of Applied Health Research Berlin
MCI: Mild cognitive impairment
OT: Occupational therapy
PWD: People with dementia
PWD/MCI: People with dementia or mild cognitive impairment
PS5/4: Indication code dementia syndromes
RECORD: Reporting of studies Conducted using Observational Routinely-collected health Data Statement
RCSLT: Royal College of Speech and Language Therapists
SC: Indication code Swallowing disorders
SGB: German Social Code (Sozialgesetzbuch)
SHI: Statutory health insurance
SLT: Speech-Language therapy
SP5: Indication code Speech disorders after completing speech development
