# Supplementary material for: Speech-language therapy and occupational therapy for patients with mild cognitive impairment and dementia: a retrospective cohort study using German health claims data
Source: BMC Health Serv Res. 2025 Aug 5;25:1026. doi: 10.1186/s12913-025-13149-y (PMC12326587; doi:10.1186/s12913-025-13149-y)
Supplement: Supplementary file 1 — Supplementary Material 1 [file 12913_2025_13149_MOESM1_ESM.docx]

**Supplementary Material**

**Supplementary Table S1:**

*The RECORD statement – checklist of items, extended from the STROBE statement*

|  | **Item No.** | **STROBE items** | **Location in manuscript where items are reported/ details** | **RECORD items** | **Location in manuscript where items are reported** |
| --- | --- | --- | --- | --- | --- |
| **Title and abstract** | | |  |  |  |
|  | 1 | (a) Indicate the study’s design with a commonly used term in the title or the abstract (b) Provide in the abstract an informative and balanced summary of what was done and what was found | Retrospective cohort study,  p.1 title  p.2. abstract | RECORD 1.1: The type of data used should be specified in the title or abstract. When possible, the name of the databases used should be included.    RECORD 1.2: If applicable, the geographic region and timeframe within which the study took place should be reported in the title or abstract.    RECORD 1.3: If linkage between databases was conducted for the study, this should be clearly stated in the title or abstract. | German Health Claims Data,  InGef Database  p.2  Objective/Methods  Germany, 2017-2022  p.2 Methods  no linkage |
| **Introduction** | | |  |  |  |
| Background rationale | 2 | Explain the scientific  background and rationale for the investigation being reported | Introduction, p. 4-6 | | |
| Objectives | 3 | State specific objectives, including any prespecified hypotheses | Introduction: Aim of the study I and II, p. 6  Methods: Study Design: no prespecified hypotheses, p. 7 | | |
| **Methods** | | |  |  |  |
| Study Design | 4 | Present key elements of study design early in the paper | Methods: Study design: p. 7  - a retrospective observational study using routinely collected claims-based data | | |
| Setting | 5 | Describe the setting, locations, and relevant dates, including periods of recruitment, exposure, follow-up, and data collection | Methods: Study Design, Data Source: p. 7-8  Methods: Population, p. 8 | | |

| Participants | 6 | 1. *Cohort study* - Give the eligibility criteria, and the sources and methods of selection of participants. Describe methods of follow-up   *Case-control study* - Give the eligibility criteria, and the sources and methods of case ascertainment and control selection. Give the rationale for the choice of cases and controls *Cross-sectional study* - Give the eligibility criteria, and the sources and methods of selection of participants     1. *Cohort study* - For matched studies, give matching criteria and number of exposed and unexposed   *Case-control study* - For matched studies, give matching criteria and the number of controls per case | Methods: Data source, population: p. 7-8 | RECORD 6.1: The methods of study population selection (such as codes or algorithms used to identify subjects) should be listed in detail. If this is not possible, an explanation should be provided.    RECORD 6.2: Any validation studies of the codes or algorithms used to select the population should be referenced. If validation was conducted for this study and not published elsewhere, detailed methods and results should be provided.    RECORD 6.3: If the study involved linkage of databases, consider use of a flow diagram or other graphical display to demonstrate the data linkage process, including the number of individuals with linked data at each stage. | Methods: population, p. 8 diagnosis following ICD-10-GM: F00-F03, F06.7  Methods: population, p. 8  Validation of diagnosis (M2Q Criteria for outpatient diagnosis)  No linkage |
| --- | --- | --- | --- | --- | --- |
| Variables | 7 | Clearly define all outcomes, exposures, predictors, potential confounders, and effect modifiers. Give diagnostic criteria, if applicable. | Methods, Measures, p. 9 | RECORD 7.1: A complete list of codes and algorithms used to classify exposures, outcomes, confounders, and effect modifiers should be provided. If these cannot be reported, an explanation should be provided. | Supplementary material, Table 2, p. 8 |
| Data sources/ measurement | 8 | For each variable of interest, give sources of data and details of methods of assessment (measurement).  Describe comparability of assessment methods if there is  more than one group | Methods, Measure, p. 9  Supplementary material, Table 2, p. 8 | | |

| Bias | 9 | Describe any efforts to address potential sources of bias | Methods, Population: p. 8 diagnostic validation | | |
| --- | --- | --- | --- | --- | --- |
| Study size | 10 | Explain how the study size was arrived at | Methods, Population: p. 8, Results: Data Flow, p. 11 | | |
| Quantitative variables | 11 | Explain how quantitative variables were handled in the analyses. If applicable, describe which groupings were chosen,  and why | Methods: Measures, p. 9 | | |
| Statistical methods | 12 | (a) Describe all statistical methods, including those used to control for confounding (b) Describe any methods used to examine subgroups and interactions   1. Explain how missing data were addressed 2. *Cohort study* - If applicable, explain how loss to follow-up was addressed   *Case-control study* - If applicable, explain how matching of cases and controls was addressed  *Cross-sectional study* - If applicable, describe analytical methods taking account of sampling strategy   1. Describe any sensitivity analyses | Methods, Analysis: p. 10 | | |
| Data access and cleaning methods |  | ..  / | | RECORD 12.1: Authors should describe the extent to which the investigators had access to the database population used to create the study population. | Analysis: p.10  All analyses were performed exclusively by InGef staff using the statistical software R, version 4.0.2 |

|  |  |  |  | RECORD 12.2: Authors should provide information on the data cleaning methods used in the study. | Analysis, p. 10 |
| --- | --- | --- | --- | --- | --- |
| Linkage |  |  |  | RECORD 12.3: State whether the study included person-level,  institutional-level, or other data linkage across two or more databases. The methods of linkage and methods of linkage quality evaluation should be provided. | Measures: p. 9  individual-level, no data linkage across two databases |
| **Results** | | | | | |
| Participants | 13 | 1. Report the numbers of individuals at each stage of the study (*e.g.*, numbers potentially eligible, examined for eligibility, confirmed eligible, included in the study, completing follow-up, and analysed) 2. Give reasons for nonparticipation at each stage. (c) Consider use of a flow diagram | Results, figure 1 data flow: p. 11 | RECORD 13.1: Describe in detail the selection of the persons included in the study (*i.e.,* study population selection) including filtering based on data quality, data availability and linkage. The selection of included persons can be described in the text and/or by means of the study flow diagram. | Results, figure 1 data flow: p. 11 |
| Descriptive data | 14 | 1. Give characteristics of study participants (*e.g.*, demographic, clinical, social) and information on exposures and potential   confounders   1. Indicate the number of participants with missing data for each variable of interest (c) *Cohort study* - summarise follow-up time (*e.g.*, average and total amount) | A). Results, Study Population: Table 1: p. 12    B) – no missing data | | |
| Outcome data | 15 | *Cohort study* - Report numbers of outcome events or summary measures over time  *Case-control study* - Report numbers in each exposure | Results, Study population, Utilisation of SLT and OT: p. 11 ongoing  - outcome: utilisation SLT/OT: number of patients with use/ number of prescriptions per patient / billet therapy sessions per patient | | |

|  |  | category, or summary measures of exposure  *Cross-sectional study* - Report numbers of outcome events or summary measures |  | | |
| --- | --- | --- | --- | --- | --- |
| Main results | 16 | (a) Give unadjusted estimates and, if applicable, confounderadjusted estimates and their precision (e.g., 95% confidence interval). Make clear which confounders were adjusted for and why they were included (b) Report category boundaries when continuous variables were categorized  (c) If relevant, consider translating estimates of relative risk into absolute risk for a meaningful time period | Mainly descriptive results.  Results: Association between utilisation and sociodemographic and clinical variables, p. 17-19  Methods: Analysis: p. 10, 95% confidence interval, (α = 0.05). | | |
| Other analyses | 17 | Report other analyses done—  e.g., analyses of subgroups and interactions, and sensitivity analyses | No other analyses done | | |
| **Discussion** | | | | | |
| Key results | 18 | Summarise key results with reference to study objectives | Discussion: Summary of findings, p. 20 | | |
| Limitations | 19 | Discuss limitations of the study, taking into account sources of potential bias or imprecision. Discuss both direction and magnitude of any potential bias | Discussion: Limitations, p. 26 | RECORD 19.1: Discuss the  implications of using data that were not created or collected to answer the specific research question(s). Include discussion of misclassification bias, unmeasured confounding, missing data, and changing eligibility over time, as they pertain to the study being reported. | Limitations: p. 26  confounding: comorbidities, duration and severity of dementia, |
| Interpretation | 20 | Give a cautious overall interpretation of results considering objectives,  limitations, multiplicity of analyses, results from similar studies, and other relevant evidence | Discussion of findings: p. 20 ongoing | | |
| Generalisability | 21 | Discuss the generalisability (external validity) of the study results | Discussion: Generalisability therapy utilisation: p. 23  Limitations: p. 26 | | |
| **Other Information** | | | | | |
| Funding | 22 | Give the source of funding and the role of the funders for the present study and, if applicable, for the original study on which the present article is based | Declaration, no competing interests.  Funding: Bochum University of Applied Sciences. Location Health Campus. Department of Nursing, Midwifery and Therapy Sciences. | | |
| Accessibility of protocol, raw data, and programming code |  | .. | RECORD 22.1: Authors should provide information on how to access any supplemental information such as the study protocol, raw data, or programming code. | | Via contact of first author |

*Reference: Benchimol EI, Smeeth L, Guttmann A, Harron K, Moher D, Petersen I, Sørensen HT, von Elm E, Langan SM, the RECORD Working Committee. The REporting of studies Conducted using Observational Routinely-collected health Data (RECORD) Statement. *PLoS Medicine* 2015; in press.

*Checklist is protected under Creative Commons Attribution ([CC BY)](http://creativecommons.org/licenses/by/4.0/) license.

**Supplementary Table S2:**

*Variables included in the analysis, their characteristics and codes used*

| **Variable** | | **Explanation** | | **Characteristics (Codes)** |
| --- | --- | --- | --- | --- |
| Socio-demographic information | | | | |
| Age | | When first diagnosed | | Age in years |
| Age in groups | | Categorised into five-year groups. When first prescribed SLT/OT | | 18-64 |
|  |  |  |  | 65-69 |
|  |  |  |  | 70-74 |
|  |  |  |  | 75-79 |
|  |  |  |  | 80-84 |
|  |  |  |  | 85-90 |
|  |  |  |  | >90 |
| Sex | | When first diagnosed/ when first prescribed SLT/OT | | Female |
|  |  |  |  | Male |
| Clinical information | | | | |
| Dementia type | | Outpatient diagnoses: at least two confirmed diagnoses, either documented by two different physicians in the same quarter or documented in two quarters Inpatient diagnoses: at least one main or secondary diagnosis at discharge (outpatient diagnosis may also be validated by an inpatient diagnosis) | | Dementia in Alzheimer disease: ICD-10-GM: F00 |
|  |  |  |  | Vascular dementia: ICD-10-GM: F01 |
|  |  |  |  | Dementia in other diseases classified elsewhere: ICD-10-GM: F02 |
|  |  |  |  | Dementia in Pick disease: ICD-10-GM: F02.0 |
|  |  |  |  | Unspecified dementia: ICD-10-GM: F03 |
|  |  |  |  | Mild cognitive impairment (MCI): ICD-10-GM: F06.7 |
| Specialty of the prescribing physician | | Specialist group of the first SLT/ OT prescription | | General Practitioner: part of the anonymized Lifelong Physician Identification Number: 01/02/03 |
|  |  |  |  | Internist (geriatric medicine): 23, 32 |
|  |  |  |  | Neurology/Psychiatry: 51 |
|  |  |  |  | Neurology: 53 |
|  |  |  |  | Psychiatry: 58 |
|  |  |  |  | No Information |
|  |  |  |  | Other |
| Utilization of nonpharmacological therapies: SLT | | | | |
| Patients with utilization of SLT (independent of indication code) | | with at least one prescription | | Number of patients with at least one prescription |
|  |  |  |  | Number of prescriptions per patient |
|  |  |  |  | Number of billed therapy sessions per patient with prescriptions |
| Utilization of nonpharmacological therapies: SLT Indication code: SP5 Speech disorders after completing speech development | | | | |
| Patients with utilization of SLT for communication impairment | | with at least one prescription | | Number of patients with at least one prescription in indication group PS5 |
|  |  |  |  | Number of prescriptions per patient in indication group SP5 |
|  |  |  |  | Number of therapy sessions per patient with prescriptions in indication group SP5 |
|  |  | With regular use: in at least three of the eight quarters of follow-up (two years) there was at least one prescription. | | Number of patients with regular prescription |
|  |  |  |  | Number of prescriptions per patient with regular use |
|  |  |  |  | Number of billed therapy sessions per patient with regular use |
|  |  | Prescribed therapeutic treatment (remedies) per prescription for indication group SP5 | | Speech therapy 30 minutes: BRN: X3102 |
|  |  |  |  | Speech therapy 45 minutes: BRN: X3103 |
|  |  |  |  | Speech therapy 60 minutes: BRN: X3104 |
|  |  |  |  | Speech therapy group treatment 45 minutes: BRN: X3220 |
|  |  |  |  | Speech therapy group treatment 90 minutes: BRN: X3223 |
|  |  | Prescription with additional home visit service | | Number of patients with at least one prescription in indication group SP5 |
|  |  |  |  | Number of prescriptions per patient in indication group SP5 |
|  |  |  |  | Number of billed therapy sessions per patient with prescriptions in indication group SP5 (BRN:X9901) |
| Utilization of nonpharmacological therapies: SLT Indication code: SC Swallowing disorders (dysphagia) | | | | |
| Patients with utilization of SLT for dysphagia | | Patients with at least one prescription | | Number of patients with at least one prescription in indication group SC |
|  |  |  |  | Number of prescriptions per patient in indication group SC |
|  |  |  |  | Number of billed therapy sessions per patient with prescriptions in indication group SC |
|  |  | With regular use: in at least three of the eight quarters of follow-up (two years) there was at least one prescription. | | Number of patients with regular prescription |
|  |  |  |  | Number of prescriptions per patient with regular use |
|  |  |  |  | Number of billed therapy sessions per patient with regular use |
|  |  | Prescribed therapeutic treatment (remedies) per prescription for indication group SC | | Speech therapy 30 minutes: BRN: X3102 |
|  |  |  |  | Speech therapy 45 minutes: BRN: X3103 |
|  |  |  |  | Speech therapy 60 minutes: BRN: X3104 |
|  |  | Prescription with additional home visit service | | Number of patients with at least one prescription in indication group SC |
|  |  |  |  | Number of prescriptions per patient in indication group SC |
|  |  |  |  | Number of billed therapy sessions per patient with prescriptions in indication group SC (BRN:X9901) |
| Utilization of nonpharmacological therapies: OT | | | | |
| Patients with utilization of OT (independent of indication code) | with at least one prescription | | Number of patients with at least one prescription | |
|  |  |  | Number of prescriptions per patient | |
|  |  |  | Number of billed therapy sessions per patient with prescriptions | |
| Utilization of nonpharmacological therapies: OT Indication code: PS5/PS4 dementia syndromes* | | | | |
| * The German Remedies Catalogue was modified in 2021. Until December 2020, the indication code for dementia syndromes was PS5. In January 2021, it was replaced by code PS4. However, the content remained unchanged. | | | | |
| Patients with utilization of OT | | Patients with at least one prescription | | Number of patients with at least one prescription in indication group PS5/4 |
|  |  |  |  | Number of prescriptions per patient in indication group PS5/4 |
|  |  |  |  | Number of billed therapy sessions per patient with prescriptions in indication group PS5/4 |
|  |  | With regular use: in at least three of the eight quarters of follow-up (two years) there was at least one prescription. | | Number of patients with regular prescription |
|  |  |  |  | Number of prescriptions per patient with regular use |
|  |  |  |  | Number of billed therapy sessions per patient with regular use |
|  |  | Prescribed therapeutic treatment (remedies) per prescription for indication group PS5/4 | | Occupational therapy cognitive training: BRN: X4104 |
|  |  |  |  | Occupational therapy cognitive training group treatment: BRN: X4211 |
|  |  |  |  | Occupational therapy psycho-functional treatment: BRN: X4105 |
|  |  |  |  | Occupational therapy psycho-functional treatment group treatment  BRN: X4212 |
|  |  | Prescription with additional home visit service | | Number of patients with at least one prescription in indication group PS5/4 |
|  |  |  |  | Number of prescriptions per patient in indication group PS5/4 |
|  |  |  |  | Number of billed therapy sessions per patient with prescriptions in indication group PS5/4 (BRN:X9933) |
| Utilization of several nonpharmacological therapies | | | | |
| Patients with utilization of several therapies | | At least one prescription per indication | | SP5 and SC  BRN: X3102 X3103 X3104 X3220 X3223 |
|  |  |  |  | SP5 and PS5/4  BRN: X3102 X3103 X3104 X3220 X3223 X4104 X4105 X4211 X4212 |
|  |  |  |  | SC and PS5/4  BRN: X3102 X3103 X3104 X4104 X4105 X4211 X4212 |
|  |  |  |  | SP5, SC and PS5/4  BRN: X3102 X3103 X3104 X3220 X3223 X4104 X4105 X4211 X4212 |

*OT: Occupational Therapy, SLT: Speech-Language-Therapy*

**Supplementary Figure S1**

*Multivariable associations between SLT for communication impairment and sociodemographic/ clinical variables (N=63,496)*

*
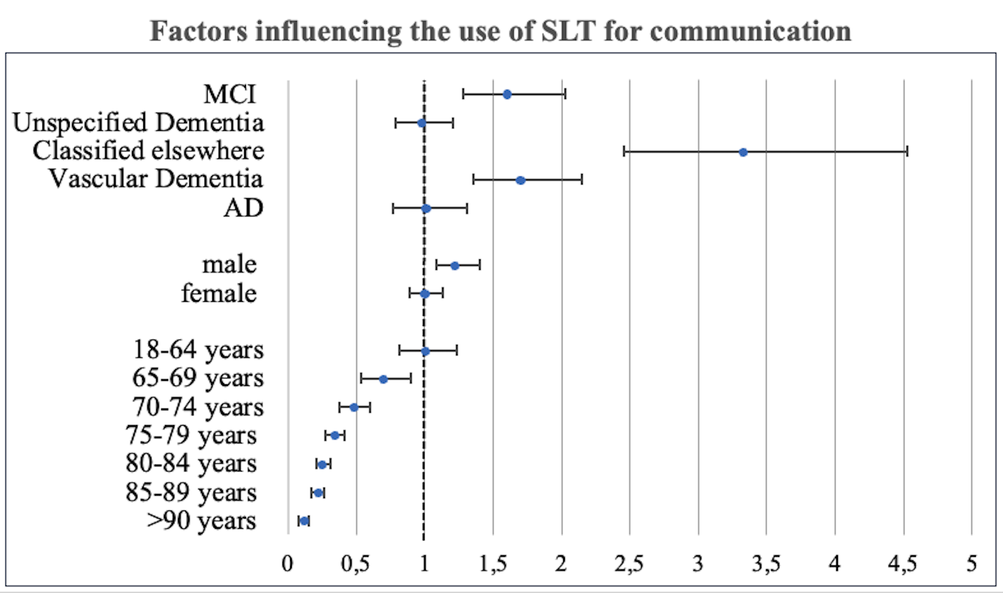
*
*Factors associated with the use of SLT for communication impairment in the study population. The forest plot displays adjusted ORs with 95% CIs for relevant socio-demographic and clinical characteristics. Reference categories: AD for dementia type, female for sex, and 18-64 years for age group. ORs >1 indicate higher likelihood of SLT use compared to the reference group. Abbreviations: AD = Alzheimer’s disease, CI = confidence interval, MCI = mild cognitive impairment, OR = odds ratio, SLT = speech-language therapy.*

**Supplementary Figure S2**

*Multivariable associations between SLT for dysphagia and sociodemographic/ clinical variables (N=63,496)*

*
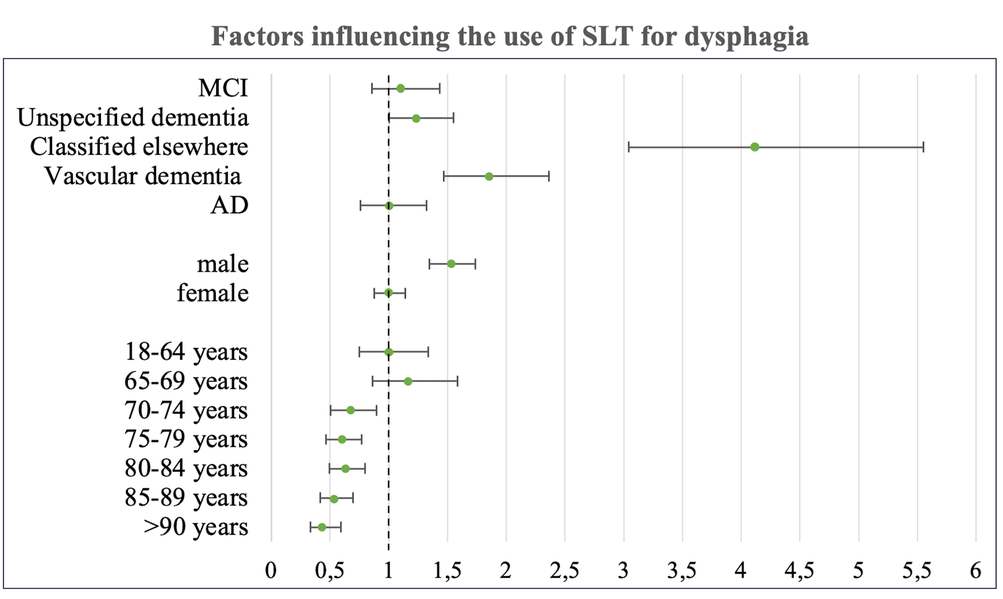
*

*Factors associated with the use of SLT for dysphagia therapy in the study population. The forest plot displays adjusted ORs with 95% CIs for relevant socio-demographic and clinical characteristics. Reference categories: AD for dementia type, female for sex, and 18-64 years for age group. ORs >1 indicate higher likelihood of SLT use compared to the reference group. Abbreviations: AD = Alzheimer’s disease, CI = confidence interval, MCI = mild cognitive impairment, OR = odds ratio, SLT = speech-language therapy.*
